# Social Communication of Maternal Immune Activation-Affected Offspring Is Improved by Si-Based Hydrogen-Producing Agent

**DOI:** 10.3389/fpsyt.2022.872302

**Published:** 2022-04-13

**Authors:** Noriyoshi Usui, Kazumasa Matsumoto-Miyai, Yoshihisa Koyama, Yuki Kobayashi, Yukiko Nakamura, Hikaru Kobayashi, Shoichi Shimada

**Affiliations:** ^1^Department of Neuroscience and Cell Biology, Graduate School of Medicine, Osaka University, Suita, Japan; ^2^United Graduate School of Child Development, Osaka University, Suita, Japan; ^3^Global Center for Medical Engineering and Informatics, Osaka University, Suita, Japan; ^4^Addiction Research Unit, Osaka Psychiatric Research Center, Osaka Psychiatric Medical Center, Osaka, Japan; ^5^Graduate School of Comprehensive Rehabilitation, Osaka Prefecture University, Osaka, Japan; ^6^Institute of Scientific and Industrial Research, Osaka University, Suita, Japan

**Keywords:** maternal immune activation (MIA), neurodevelopmental disorders (NDDs), autism spectrum disorder (ASD), ultrasonic vocalization (USV), social communication, inflammation, silicon, hydrogen

## Abstract

Maternal immune activation (MIA) is triggered by infection or autoimmune predisposition during pregnancy, and cytokines produced by MIA are transmitted through the placenta to the fetal brain, implicating at the onset risks and vulnerability for developmental and psychiatric disorders, such as autism spectrum disorder (ASD) and schizophrenia. To address these kinds of problem in child health, we have developed a silicon (Si)-based hydrogen-producing antioxidant (Si-based agent) that continuously and effectively produces hydrogen in the body. Medical hydrogen is known to have antioxidative, anti-inflammatory, and antiapoptotic effects, therefore we applied our Si-based agent as a potential therapeutic agent to MIA. Using a MIA mouse model, we found that the Si-based agent improved the social communication of MIA offspring mice. We also found that the Si-based agent suppressed the expressions of inflammation-associated genes *Ifna1* and *Il-6* in the mouse brain. These results demonstrate that the Si-based agent is an effective prophylactic agent against MIA during pregnancy, suggesting that our Si-based agent may be a preventative or therapeutic agent for ASD and other disease risks in child health suppressing MIA damage.

## Introduction

Maternal immune activation (MIA) is an inflammatory response triggered by a pathogenic infection and autoimmune diseases in the mother. MIA caused by infections during pregnancy increases the risks of stillbirth and miscarriage as well as the risks of neurodevelopmental disorders (NDDs) and psychiatric disorders, such as autism spectrum disorder (ASD) and schizophrenia, in children (1–4). ASD is a NDD characterized by social communication deficits, repetitive behaviors, and hyperesthesia/hypesthesia. The prevalence of ASD has been reported as 1 in 54 (1.85%) in the US (5). MIA is a well-known environmental factor to increase the risk for the onset of ASD in offspring. Infections of SARS-CoV-2 during pregnancy is the onset risk of MIA and alter maternal and fetal immune responses (6). Therefore, in the present days, there is a demand for preventive drugs that protect both mother and fetus from the influences of MIA safely and without side effects.

Medical hydrogen exerts antioxidative, anti-inflammatory, antiallergic, and antiapoptotic effects (7–10). Hydrogen selectively reduces hydroxyl radicals (•OH) in reactive oxygen species and reacts only with hydroxyl radicals. Consequently, they can be used as a therapeutic agent for diseases associated with oxidative stress and inflammation, without side effects (11, 12). Recently, we have developed a Si-based agent that can continually produce a large amount of hydrogen (up to 400 ml/g) by reaction with water under conditions (pH 8.3 and 36°C) similar to those in the gut (13–15). Si and its reaction product SiO_2_ are known to be non-toxic, enabling the oral administration of the Si-based agent. Recently, we have reported the protective effects of enteric hydrogen generated from a Si-based agent in model animals of maternal–fetal transmission, Parkinson's disease, and chronic kidney disease (15, 16). Since hydrogen is produced in the gastrointestinal system, it is physically and easily delivered to the uterus and fetus. Therefore, our Si-based agent may prevent MIA-induced health risks in the offspring.

In this study, we evaluated the preventative effect of our Si-based agent on MIA. As a mouse model of ASD, we induced MIA by polyinosinic–polycytidylic acid [poly(I:C)] injection into pregnant mother mice. The social communication of MIA offspring was assessed by ultrasonic vocalizations (USVs). We found that the Si-based agent improved the social communication of MIA offspring and inhibited inflammatory gene expressions. These results suggest the possibility of the Si-based agent as a preventative drug for MIA.

## Methods

### Mice

All procedures were performed according to the ARRIVE guidelines and relevant official guidelines under the approval (#27-010) of the Animal Research Committee of Osaka University. C57BL/6J (Japan SLC Inc., Shizuoka, Japan) pregnant female mice were used. For embryo staging, the day of detection of the vaginal plug was considered embryonic day (E) 0.5. The mice were housed in groups of 2 to 3 animals per cage (143 mm × 293 mm × 148 mm) in the barrier facilities of Osaka University under a 12-h light–dark cycle and given free access to water and food. An experimenter blinded to the group setting performed all the tests.

### Si-Based Agent and Treatment

The Si-based agent and Si-based-agent-containing feed were prepared as described previously (15, 16). A Si-based agent was produced from polycrystalline Si powder (Osaka Titanium Technologies Co., Ltd., Osaka, Japan; Si 4Nup). After milling the Si powder, surface treatment and aggregation were carried out. Therefore, the Si-based agent was composed of an aggregate of Si nanopowder. For control laboratory chow, the AIN-93M diet (Oriental Yeast Co., Ltd., Tokyo, Japan) was used. For Si-based agent-containing laboratory chow, special laboratory chow was made containing 2.5 weight% Si-based agent in AIN-93M. The feed was given to pregnant mothers starting at E8.5 until postnatal day (P) 7, with free access to food and water. Before the animal experiments, hydrogen production from feed and water was evaluated using a sensor gas chromatograph, SGHA-PA (FIS Inc., Hyogo, Japan).

### Poly(I:C) Administration

Poly(I:C) was administered as described previously (17). Briefly, 20 mg/kg poly(I:C) (#P9582; Merck, Darmstadt, Germany) dissolved in saline (5 ml/kg) (#3311401A2026; Otsuka Pharmaceutical Co., Ltd., Tokyo, Japan) was intraperitoneally injected at E12.5 or 7 weeks of female mice. Saline was used for the control group.

### USVs

USV analysis was performed as described previously (18, 19). The assessment of USVs was carried out at P7. Pups were removed from the dam and placed in individual soundproof chambers. Recordings were acquired for 3 min using UltraSoundGate condenser microphones (CM16, Avisoft Bioacoustics, Glienicke/Nordbahn, Germany) positioned at a fixed height of 20 cm above the pups and then amplified and digitized using UltraSoundGate 416H hardware and Avisoft RECORDER software (Avisoft Bioacoustics, Glienicke/Nordbahn, Germany; approximately 20 dB gain, sampled at 16 bits, 250 kHz). Sound spectrograms were prepared in MATLAB (50% overlapping, 512-point Hamming windows), resulting in 1.024-ms temporal resolution and 488.3-Hz spectral resolution. The spectrograms were band-pass-filtered to 20–120 kHz and filtered for white noise. The quantification and analysis of data included the number and duration (ms) of calls, the number of calls with/without frequency jumps, the mean frequency (kHz), the frequency range (kHz) of calls, and the mean call slope (Hz/ms). The positions of USVs were determined automatically by a previously published method (20). The features of vocalization were considered independently.

### Quantitative Real-Time PCR

Quantitative real-time PCR (qPCR) was performed as described previously (16, 21). Total RNA was extracted from the mouse prefrontal cortex (PFC) using the miRNeasy Mini Kit (#217004; Qiagen, Hilden, Germany) according to the manufacturer's instructions. Single-stranded cDNA was prepared using DNaseI, Amplification Grade (#18068015; Thermo Fisher Scientific) and SuperScript III First-Strand Synthesis SuperMix (#18080400; Thermo Fisher Scientific) and amplified by PCR according to the manufacturer's instructions. qRT-PCR was performed using PowerUp SYBR Green Master Mix (#A25742; Thermo Fisher Scientific) and a QuantStudio 7 Flex Real-Time PCR System (Thermo Fisher Scientific). Each biological sample had four technical replicates for qPCR, and the number of biological replicates for each experiment is indicated in each figure legend. *18S* rRNA was used as a reference for normalization. Data were analyzed by the ΔΔCq method using QuantStudio 7 Flex Real-Time PCR System software (Thermo Fisher Scientific). The following primers were used: *18S rRNA*, F-5′-GAGGGAGCCTGAGAAACGG-3′, R-5′-GTCGGGAGTGGGTAATTTGC-3′; *Ifna1*, F-5′-AGTGAGCTGACCCAGCAGAT-3′, R-5′-GGTGGAGGTCATTGCAGAAT-3′; *Il6*, F-5′- CTTCCATCCAGTTGCCTTCTTG-3′, R-5′- AATTAAGCCTCCGACTTGTGAAG-3′; *Il1b*, F-5′-TACAGGCTCCGAGATGAACA-3′, R-5′-AGGCCACAGGTATTTTGTCG-3′; *Tnf*, F-5′-CCACCACGCTCTTCTGTCTA-3′, R-5′-AGGGTCTGGGCCATAGAACT-3′; *Nfe2l2*, F-5′- GCTTTTGGCAGAGACATTCC-3′, R-5′-CCAAACTTGCTCCATGTCCT-3′; *Keap1*, F-5′-ATGGCCACATCTACGCAGTC-3′, R-5′- CCAATCCTCCGTGTCAACAT-3′; *Hmox1*, F-5′-GCCACCAAGGAGGTACACAT-3′, R-5′-CTTCCAGGGCCGTGTAGATA-3′; *Nqo1*, F-5′-GAAGCTGCAGACCTGGTGAT-3′, R-5′-GTTGTCGTACATGGCAGCAT-3′.

### Statistical Analysis

All data are presented as means of biological independent experiments ± standard error of the mean (SEM). Statistical analysis (two-way ANOVA) was performed using Prism 9. *P* < 0.05 was considered to indicate statistical significance.

## Results

### Si-Based Agent-Protected MIA Offspring

**Figure 1:**
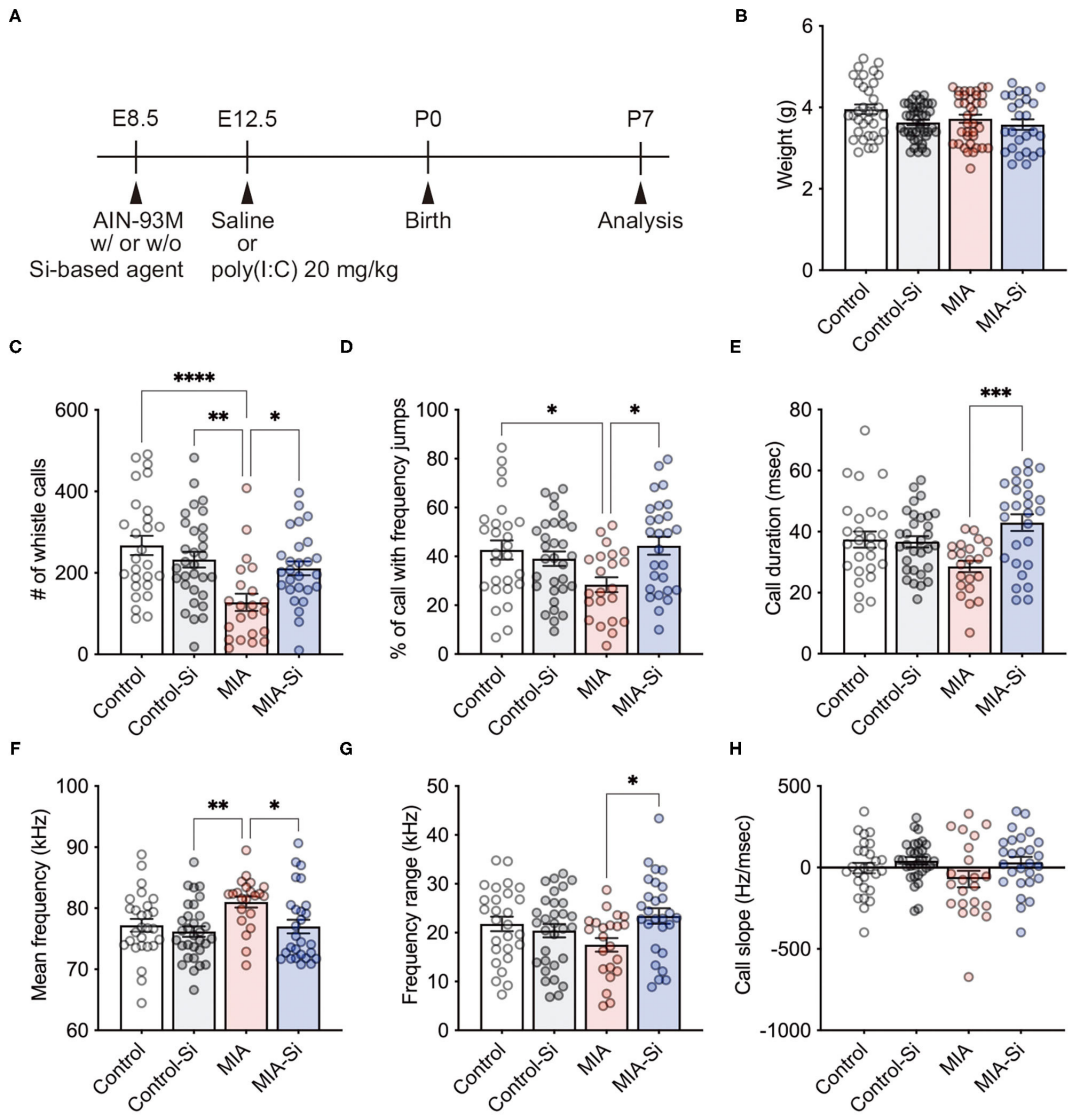
Silicon-based agent improved social communication of MIA offspring. **(A)** Experimental scheme. Pregnant mice were fed the AIN-93M diet with or without 2.5 weight% Si-based agent from E8.5 until P7. Saline or poly(I:C) (20 mg/kg) was intraperitoneally injected into pregnant mice at E12.5. **(B)** No change in the weight (interaction: *F* = 0.82, DF = 1, *P* = 0.37; MIA: *F* = 2.02, DF = 1, *P* = 0.0205; Si: *F* = 5.50, DF = 1, *P* = 0.16). **(C–H)** Mouse ultrasonic vocalizations (USVs) in the MIA offspring. The number of whistle calls (interaction: *F* = 8.24, DF = 1, *P* = 0.0050; MIA: *F* = 15.37, DF = 1, *P* = 0.24; Si: *F* = 1.40, DF = 1, *P* = 0.0002) (**C**), % of call with frequency jumps (interaction: *F* = 7.90, DF = 1, *P* = 0.0059; MIA: *F* = 1.65, DF = 1, *P* = 0.08; Si: *F* = 3.16, DF = 1, *P* = 0.20) **(D)**, call duration (ms) (interaction: *F* = 10.26, DF = 1, *P* = 0.0018; MIA: *F* = 0.30, DF = 1, *P* = 0.0044; Si: *F* = 8.47, DF = 1, *P* = 0.58) **(E)**, mean frequency (kHz) (interaction: *F* = 2.25, DF = 1, *P* = 0.14; MIA: *F* = 5.41, DF = 1, *P* = 0.0127; Si: *F* = 6.43, DF = 1, *P* = 0.0220) **(F)**, frequency range (kHz) (interaction: *F* = 6.12, DF = 1, *P* = 0.0150; MIA: *F* = 0.19, DF = 1, *P* = 0.13; Si: *F* = 2.35, DF = 1, *P* = 0.66) **(G)**, and call slope (Hz/ms) (interaction: *F* = 0.71, DF = 1, *P* = 0.40; MIA: *F* = 1.27, DF = 1, *P* = 0.0342; Si: *F* = 4.61, DF = 1, *P* = 0.26) **(H)** were analyzed. In the MIA offspring, reduced whistle calls and calls with frequency jumps and increased mean frequency were observed, but those alterations in USVs were improved with a silicon (Si)-based agent, respectively. Si, Si-based agent; MIA, maternal immune activation. Data are presented as means (±SEM). ****P < 0.0001, ***P < 0.001, **P < 0.01, *P < 0.05, one-way ANOVA with a Tukey's multiple-comparison test. *n* = 26–41/condition for weight, *n* = 22–31/condition from USVs.

To evaluate the effects of Si-based agent on MIA, we used a mouse model of MIA induced by poly(I:C) injection during pregnancy. Pregnant mother mice were fed the AIN-93M diet with or without Si-based agent from E8.5 and intraperitoneally injected saline or poly(I:C) at E12.5, and then the MIA offspring was analyzed at P7 (Figure 1A). We conducted MIA to generate a mouse model of ASD (17, 22), and investigated the social communication of MIA offspring using mouse USVs. USVs are indicators of the vocal communication between the mother and the offspring (23, 24). USVs were measured when the pups were isolated from the mother mouse.

We observed no differences in the weight of the offspring at P7 (control = 3.96 ± 0.12, control-Si = 3.63 ± 0.07, MIA = 3.72 ± 0.10, MIA-Si = 3.58 ± 0.13) (Figure 1B). We first found the significant reduction of whistle calls (control = 267.3 ± 23.72, control-Si = 232.6 ± 19.26, MIA = 127.6 ± 21.05, MIA-Si = 211.0 ± 17.05) (Figure 1C), call with frequency jumps (control = 42.63 ± 3.91, control-Si = 39.04 ± 2.98, MIA = 28.42 ± 3.04, MIA-Si = 44.34 ± 3.67) (Figure 1D), and call duration (control = 37.41 ± 2.66, control-Si = 36.72 ± 1.83, MIA = 28.60 ± 1.87, MIA-Si = 42.95 ± 2.73) (Figure 1E) in MIA offspring, but these reductions of USVs were recovered with the Si-based agent (Figures 1C–E). We also found an increased mean of frequency in MIA offspring (control = 77.21 ± 1.04, control-Si = 76.18 ± 0.86, MIA = 81.02 ± 0.90, MIA-Si = 77.00 ± 1.22) (Figure 1F), but it was also recovered with the Si-based agent (Figure 1F). We further observed the improvement of frequency range with the Si-based agent (control = 21.80 ± 1.47, control-Si = 20.41 ± 1.36, MIA = 17.50 ± 1.40, MIA-Si = 23.42 ± 1.59) (Figure 1G). There was no difference in the call slope (control = −3.25 ± 32.02, control-Si = 41.40 ± 22.84, MIA = −70.84 ± 50.82, MIA-Si = 31.70 ± 33.64) (Figure 1H). These results indicate that the Si-based agent improves the social communication of MIA offspring and protects them from MIA.

### Si-Based Agent Suppressed the Inflammatory Gene Expressions

Next, we evaluated the effects of the Si-based agent on poly(I:C)-induced inflammation. The female mouse PFC was dissected at 6 h after the poly(I:C) injection, and the expressions of inflammatory and antioxidant system-related genes were investigated. Since poly(I:C) induces the expressions of inflammation-associated genes such as *Il6* and *Tnf* (17), we investigated the anti-inflammatory effects of the Si-based agent on the MIA mouse brain.

**Figure 2:**
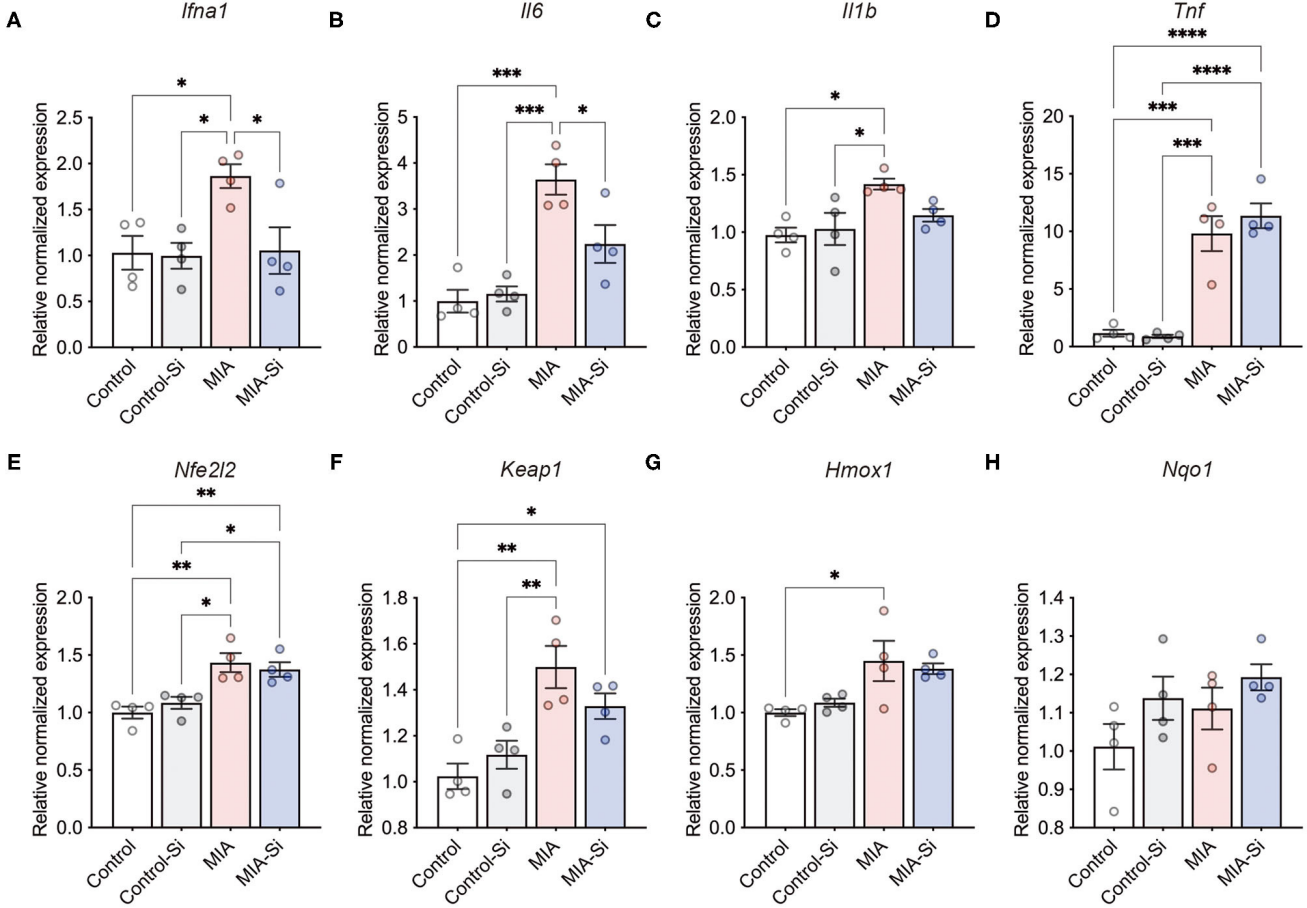
Effects of Si-based agent on inflammatory and antioxidant system-related gene expressions. **(A–D)** Relative expressions of inflammatory genes (*Ifna1, Il6, Il1b*, and *Tnf*) in the mouse prefrontal cortex (PFC) at 6 h after poly(I:C) injection. Inflammatory gene expressions were increased by poly(I:C) injection. In contrast, poly(I:C)-induced expressions of *Ifna1* (interaction: *F* = 4.48, DF = 1, *P* = 0.06; MIA: *F* = 5.91, DF = 1, *P* = 0.0403; Si: *F* = 5.29, DF = 1, *P* = 0.0317) and *Il6* (interaction: *F* = 6.65, DF = 1, *P* = 0.0242; MIA: *F* = 38.10, DF = 1, *P* = 0.06; Si: *F* = 4.24, DF = 1, *P* < 0.0001) were improved with a Si-based agent, but not in *Il1b* (interaction: *F* = 3.61, DF = 1, *P* = 0.08; MIA: *F* = 10.87, DF = 1, *P* = 0.22; Si: *F* = 1.64, DF = 1, *P* = 0.0064) and *Tnf* (interaction: *F* = 0.94, DF = 1, *P* = 0.35; MIA: *F* = 103.0, DF = 1, *P* = 0.52; Si: *F* = 0.45, DF = 1, *P* < 0.0001) expressions. **(E–H)** Relative expression levels of antioxidant system-related genes [*Nfe2l2* (interaction: *F* = 1.27, DF = 1, *P* = 0.28; MIA: *F* = 31.82, DF = 1, *P* = 0.84; Si: *F* = 0.04, DF = 1, *P* = 0.0001), *Keap1* (interaction: *F* = 3.81, DF = 1, *P* = 0.07; MIA: *F* = 25.87, DF = 1, *P* = 0.58; Si: *F* = 0.32, DF = 1, *P* = 0.003), *Hmox1* (interaction: *F* = 0.68, DF = 1, *P* = 0.43; MIA: *F* = 15.87, DF = 1, *P* = 0.92; Si: *F* = 0.01, DF = 1, *P* = 0.0018), and *Nqo1* (interaction: *F* = 0.19, DF = 1, *P* = 0.67; MIA: *F* = 2.21, DF = 1, *P* = 0.07; Si: *F* = 4.01, DF = 1, *P* = 0.16)] in the mouse PFC at 6 h after poly(I:C) injection. Si, Si-based agent; MIA, maternal immune activation. Data are presented as means (±SEM). ****P < 0.0001, ***P < 0.001, **P < 0.01, *P < 0.05, one-way ANOVA with a Tukey's multiple-comparison test. *n* = 4/condition.

We examined the expressions of inflammation-associated genes using qPCR and found that the Si-based agent suppressed *Ifna1* (control = 1.00 ± 0.18, control-Si = 1.00 ± 0.14, MIA = 1.86 ± 0.13, MIA-Si = 1.05 ± 0.25) and *Il6* (control = 1.00 ± 0.25, control-Si = 1.15 ± 0.17, MIA = 3.64 ± 0.33, MIA-Si =2.24 ± 0.41) expressions in the mouse PFC (Figures 2A,B). We also found that there was no change in *Il1b* (control = 1.00 ± 0.06, control-Si = 1.03 ± 0.14, MIA = 1.42 ± 0.05, MIA-Si = 1.15 ± 0.05, *F* = 5.38, *P* = 0.0141) and *Tnf* (control =1.00 ± 0.29, control-Si = 0.88 ± 0.15, MIA = 9.81 ± 1.51, MIA-Si = 11.35 ± 1.08) expressions (Figures 2C,D). About *Il1b* expression, there was no statistical difference, but it tended to improve (MIA = 1.42 ± 0.05, MIA-Si = 1.15 ± 0.05, 95%CI = −0.09 to 0.63, *P* = 0.16) (Figure 2C). In contrast to inflammation-associated genes, we found that there were no differences in the antioxidant system-related genes with/without Si-based agent in MIA (*Nfe2l2*: control = 1.00 ± 0.05, control-Si = 1.09 ± 0.05, MIA = 1.43 ± 0.08, MIA-Si = 1.37 ± 0.06; *Keap1*: control = 1.00 ± 0.06, control-Si = 1.12 ± 0.06, MIA = 1.50 ± 0.09, MIA-Si = 1.33 ± 0.06; *Hmox1*: control = 1.00 ± 0.06, control-Si = 1.12 ± 0.06, MIA = 1.50 ± 0.09, MIA-Si = 1.33 ± 0.06; *Nqo1*: control = 1.00 ± 0.06, control-Si = 1.14 ± 0.06, MIA = 1.11 ± 0.05, MIA-Si = 1.19 ± 0.03) (Figure 2E–**H**). These results indicate that the Si-based agent acts as an anti-inflammatory agent at an early phase of inflammation.

## Discussion

In this study, we demonstrate the effects of our Si-based agent on MIA. The Si-based agent improved the social communication of MIA offspring and suppressed the expressions of inflammatory genes. Our results suggest that the Si-based agent can be used for preventing MIA during pregnancy as a preventative agent.

MIA is one of the major environmental factors in the onset of ASD. Many studies have been reported regarding inflammation in ASD as a risk factor (3), which is also closely related to oxidative stress (25). MIA triggers maternal inflammation, and Th17 cells activated by IL-6 secrete IL-17a. Maternally derived IL-17a crosses the placenta and induces neuronal cell death by acting on IL-17a receptors expressed in the fetal brain (17). It is thought that this cell death causes a decrease in PV-positive GABAergic neurons in the cortex (22), resulting in the disturbance of E/I balance and leading to the pathogenesis of ASD (26).

MIA also dysregulates ASD-associated genes and neurodevelopmental genes (27–30)—for example, MIA downregulates the genes involved in axonal guidance, neurogenesis, and cytoskeleton, while MIA upregulates the genes involved in translation, cell cycle, and DNA damage (27). MIA also induces behavioral abnormalities, including ASD-like behaviors in mice, such as social, anxiety-like, stereotypic, and sensorimotor gating behaviors with volume of the dorsal and ventral hippocampus and anterior cingulate cortex (28). According to the latest single-cell transcriptome analysis, the genes of the mouse embryonic brain affected by MIA include those involved in mRNA translation, ribosome biogenesis, and stress signals, resulting in reduced global mRNA translation and altered nascent proteome synthesis (30).

The inhibition of inflammation within the first term in MIA is key to prevent the onset risk of ASD. In this study, we found that the Si-based agent inhibited *Il6* expression at 6 h after poly(I:C) injection (Figure 2), indicating that the Si-based agent plays a role in anti-inflammatory effects in the body. We have also reported that the Si-based agent inhibited lipopolysaccharide (LPS)-induced systemic inflammation model-induced IL-6 expression in the placenta of a mouse model of maternal–fetal transmission (16). IFN-α is produced by leukocytes activated by viruses and cytokines and plays a role in antiviral activity, immune regulation, growth inhibition, and apoptosis induction (31, 32). IL-6 acts not only on the immune system but also on many physiological events, such as regulation of cell proliferation, gene activation, growth, survival, and differentiation (33, 34). IL-1β is a potent pro-inflammatory cytokine that is induced by lymphocytes, macrophages, and monocytes and stimulates CD4+ cells to differentiate into Th17 cells (35, 36). TNF-α plays a role in the inflammatory response locally and in the blood and is essential for the early response to viral infection by promoting lymphocyte infiltration (37–39). Our results showed that the Si-based agent had an effect on some inflammatory cytokines' gene expression and no effect on some others (Figure 2). However, only one shot after inflammation was analyzed, and the effect of the Si-based agent on the time course of inflammation was not examined. Our results together suggest that the Si-based agent can protect the fetus from MIA by suppressing inflammation.

In this study, we speculate that one of the reasons why an effect was not observed on the expression of antioxidant system-related genes was due to the early stages of inflammation (Figure 2). A previous study has reported that *Keap1* expression was increased in the hippocampus of MIA mice at postnatal days 28 (P28), but not in the PFC (40). The discrepancy between their finding and ours can be explained by the experimental design and methodology. They injected a low amount (5 mg/kg/day) of poly(I:C) for six times and collected the brain to examine the expression of *Keap1* mRNA at 28 days after the poly(I:C) administration. In contrast, we injected 20 mg/kg of poly(I:C) once, then at 6 h after poly(I:C) injection, we collected the brain and examined *Keap1* mRNA expression in the PFC. Since we examined the gene expression only 6 h after the poly(I:C) injection, it is possible that the effects of oxidative stress were not severe. However, our previous studies have demonstrated that the Si-based agent exhibits an antioxidant activity in the animal models of several disorders (15, 16). In a future study, we would like to demonstrate the effect of the Si-based agent on oxidative stress caused by MIA in medium- and long-term phases.

We acknowledge several limitations in this study. Although we demonstrate the preventative effects of the Si-based agent on maternal–fetal transmission (16) and MIA, the detailed molecular mechanisms of the Si-based agent are largely unknown. There is also a need to investigate the effect of the Si-based agent after MIA and show its potential as a therapeutic agent for MIA or ASD—for example, it is an interesting issue from a clinical standpoint whether the symptom-improving effects on social communication and repetitive behaviors can be observed in MIA-induced ASD model mice. In addition, we examined the anti-inflammatory effects of the Si-based agent in female mice as a model. Thus, the anti-inflammatory effects in MIA should be investigated in pregnant mother mice. Lastly, testing the safety of the long-term administration of the Si-based agent and its administration during pregnancy is an issue that should be carefully considered in animal models and clinical trials.

Overall, the Si-based agent improved the symptoms of MIA offspring in social communication and anti-inflammation. In the study of the mouse group showing resistance to MIA, the inflammatory cytokines in plasma did not increase and were similar to those in the control group (29). The prophylactic effects of our Si-based agent may be effective in supporting such resilience to MIA. In conclusion, our study provides the possibility of the Si-based agent as a preventative agent for MIA.

## Data Availability Statement

The original contributions presented in the study are included in the article/supplementary material, further inquiries can be directed to the corresponding authors.

## Ethics Statement

The animal study was reviewed and approved by the Animal Research Committee of Osaka University.

## Author Contributions

NU contributed to conceptualization, methodology, validation, investigation, writing—original draft, writing—review and editing, project administration, and funding acquisition. KM-M, YKoy, and YN contributed to investigation. YKob contributed to investigation and resources. HK contributed to resources, writing—review and editing, supervision, and funding acquisition. SS contributed to writing—review and editing, supervision, project administration, and funding acquisition. All authors contributed to the article and approved the submitted version.

## Funding

This work was supported by the Japan Science and Technology Agency (JST) Center of Innovation Program (COI Program) (JPMJCE1310) to NU, HK, and SS, the Japan Society for the Promotion of Science (JSPS) Grant-in-Aid for Scientific Research (C) (20K06872) to NU, JSPS Grant-in-Aid for Early-Career Scientists (18K14814) to NU, JSPS Grant-in-Aid for Challenging Research (20K21654) to NU and SS, Uehara Memorial Foundation to NU, Takeda Science Foundation to NU, SENSHIN Medical Research Foundation to NU, Osaka Medical Research Foundation for Intractable Diseases to NU, Public Health Science Foundation to NU, and Eli Lilly Japan Research Grant to NU. Eli Lilly was not involved in the study design, collection, analysis, interpretation of data, the writing of this article or the decision to submit it for publication.

## Conflict of Interest

The authors declare that the research was conducted in the absence of any commercial or financial relationships that could be construed as a potential conflict of interest.

## Publisher's Note

All claims expressed in this article are solely those of the authors and do not necessarily represent those of their affiliated organizations, or those of the publisher, the editors and the reviewers. Any product that may be evaluated in this article, or claim that may be made by its manufacturer, is not guaranteed or endorsed by the publisher.

## References

[1] Estes ML McAllister AK. Maternal immune activation: implications for neuropsychiatric disorders. Science. (2016) 353:772–7. 10.1126/science.aag3194 27540164 PMC5650490

[2] Meyer U. Neurodevelopmental resilience and susceptibility to maternal immune activation. Trends Neurosci. (2019) 42:793–806. 10.1016/j.tins.2019.08.001 31493924

[3] Han VX Patel S Jones HF Dale RC. Maternal immune activation and neuroinflammation in human neurodevelopmental disorders. Nat Rev Neurol. (2021) 17:564–79. 10.1038/s41582-021-00530-8 34341569

[4] Gumusoglu SB Stevens HE. Maternal inflammation and neurodevelopmental programming: a review of preclinical outcomes and implications for translational psychiatry. Biol Psychiatry. (2019) 85:107–21. 10.1016/j.biopsych.2018.08.008 30318336

[5] Maenner MJ Shaw KA Baio J Washington A Patrick M DiRienzo M. Prevalence of autism spectrum disorder among children aged 8 years - autism and developmental disabilities monitoring network, 11 sites, United States, 2016. MMWR Surveill Summ. (2020) 69:1–12. 10.15585/mmwr.ss6904a1 32214087 PMC7119644

[6] Garcia-Flores V Romero R Xu Y Theis KR Arenas-Hernandez M Miller D. Maternal-fetal immune responses in pregnant women infected with SARS-CoV-2. Nat Commun. (2022) 13:320. 10.1038/s41467-021-27745-z 35042863 PMC8766450

[7] Ohsawa I Ishikawa M Takahashi K Watanabe M Nishimaki K Yamagata K. Hydrogen acts as a therapeutic antioxidant by selectively reducing cytotoxic oxygen radicals. Nat Med. (2007) 13:688–94. 10.1038/nm1577 17486089

[8] Ohta S. Molecular hydrogen as a preventive and therapeutic medical gas: initiation, development and potential of hydrogen medicine. Pharmacol Ther. (2014) 144:1–11. 10.1016/j.pharmthera.2014.04.006 24769081

[9] Shigeo O. Recent progress toward hydrogen medicine: potential of molecular hydrogen for preventive and therapeutic applications. Curr Pharm Design. (2011) 17:2241–52. 10.2174/138161211797052664 21736547 PMC3257754

[10] Ohta S. Molecular hydrogen is a novel antioxidant to efficiently reduce oxidative stress with potential for the improvement of mitochondrial diseases. Biochim Biophys Acta General Subj. (2012) 1820:586–94. 10.1016/j.bbagen.2011.05.006 21621588

[11] Fontanari P Badier M Guillot C Tomei C Burnet H Gardette B. Changes in maximal performance of inspiratory and skeletal muscles during and after the 7.1-MPa Hydra 10 record human dive. Euro J Appl Physiol. (2000) 81:325–8. 10.1007/s004210050050 10664092

[12] Abraini JH Gardette-Chauffour MC Martinez E Rostain JC Lemaire C. Psychophysiological reactions in humans during an open sea dive to 500 m with a hydrogen-helium-oxygen mixture. J Appl Physiol. (1994) 76:1113–8. 10.1152/jappl.1994.76.3.1113 8005852

[13] Kobayashi Y Matsuda S Imamura K Kobayashi H. Hydrogen generation by reaction of Si nanopowder with neutral water. J Nanopart Res. (2017) 19:176. 10.1007/s11051-017-3873-z 28579914 PMC5434163

[14] Imamura K Kobayashi Y Matsuda S Akai T Kobayashi H. Reaction of Si nanopowder with water investigated by FT-IR and XPS. AIP Advances. (2017) 7:085310. 10.1063/1.4989794

[15] Kobayashi Y Imamura R Koyama Y Kondo M Kobayashi H Nonomura N. Renoprotective and neuroprotective effects of enteric hydrogen generation from Si-based agent. Sci Rep. (2020) 10:5859. 10.1038/s41598-020-62755-9 32246095 PMC7125117

[16] Usui N Togawa S Sumi T Kobayashi Y Koyama Y Nakamura Y. Si-Based hydrogen-producing nanoagent protects fetuses from miscarriage caused by mother-to-child transmission. Front Med Technol. (2021) 3:665506. 10.3389/fmedt.2021.665506 35047922 PMC8757766

[17] Choi GB Yim YS Wong H Kim S Kim H Kim SV. The maternal interleukin-17a pathway in mice promotes autism-like phenotypes in offspring. Science. (2016) 351:933–9. 10.1126/science.aad0314 26822608 PMC4782964

[18] Usui N Co M Harper M Rieger MA Dougherty JD Konopka G. Sumoylation of FOXP2 regulates motor function and vocal communication through purkinje cell development. Biol Psychiatry. (2017) 81:220–30. 10.1016/j.biopsych.2016.02.008 27009683 PMC4983264

[19] Usui N Araujo DJ Kulkarni A Co M Ellegood J Harper M. Foxp1 regulation of neonatal vocalizations via cortical development. Genes Dev. (2017) 31:2039–55. 10.1101/gad.305037.117 29138280 PMC5733496

[20] Holy TE Guo Z. Ultrasonic songs of male mice. PLoS Biol. (2005) 3:e386. 10.1371/journal.pbio.0030386 16248680 PMC1275525

[21] Usui N Ono Y Aramaki R Berto S Konopka G Matsuzaki H. Early life stress alters gene expression and cytoarchitecture in the prefrontal cortex leading to social impairment and increased anxiety. Front Genet. (2021) 12:754198. 10.3389/fgene.2021.754198 34795694 PMC8593203

[22] Shin Yim Y Park A Berrios J Lafourcade M Pascual LM Soares N. Reversing behavioural abnormalities in mice exposed to maternal inflammation. Nature. (2017) 549:482–7. 10.1038/nature23909 28902835 PMC5796433

[23] Portfors CV Perkel DJ. The role of ultrasonic vocalizations in mouse communication. Curr Opin Neurobiol. (2014) 28:115–20. 10.1016/j.conb.2014.07.002 25062471 PMC4177333

[24] Takahashi T Okabe S Broin P Nishi A Ye K Beckert MV. Structure and function of neonatal social communication in a genetic mouse model of autism. Mol Psychiatry. (2016) 21:1208–14. 10.1038/mp.2015.190 26666205 PMC4909589

[25] Parker W Hornik CD Bilbo S Holzknecht ZE Gentry L Rao R. The role of oxidative stress, inflammation and acetaminophen exposure from birth to early childhood in the induction of autism. J Int Med Res. (2017) 45:407–38. 10.1177/0300060517693423 28415925 PMC5536672

[26] de la Torre-Ubieta L Won H Stein JL Geschwind DH. Advancing the understanding of autism disease mechanisms through genetics. Nat Med. (2016) 22:345–61. 10.1038/nm.4071 27050589 PMC5072455

[27] Lombardo MV Moon HM Su J Palmer TD Courchesne E Pramparo T. Maternal immune activation dysregulation of the fetal brain transcriptome and relevance to the pathophysiology of autism spectrum disorder. Mol Psychiatry. (2018) 23:1001–13. 10.1038/mp.2017.15 28322282 PMC5608645

[28] Guma E Bordignon PDC Devenyi GA Gallino D Anastassiadis C Cvetkovska V. Early or late gestational exposure to maternal immune activation alters neurodevelopmental trajectories in mice: an integrated neuroimaging, behavioral, and transcriptional study. Biol Psychiatry. (2021) 90:328–41. 10.1016/j.biopsych.2021.03.017 34053674

[29] Mueller FS Scarborough J Schalbetter SM Richetto J Kim E Couch A. Behavioral, neuroanatomical, and molecular correlates of resilience and susceptibility to maternal immune activation. Mol Psychiatry. (2021) 26:396–410. 10.1038/s41380-020-00952-8 33230204 PMC7850974

[30] Kalish BT Kim E Finander B Duffy EE Kim H Gilman CK. Maternal immune activation in mice disrupts proteostasis in the fetal brain. Nat Neurosci. (2021) 24:204–13. 10.1038/s41593-020-00762-9 33361822 PMC7854524

[31] McNab F Mayer-Barber K Sher A Wack A O'Garra A. Type I interferons in infectious disease. Nat Rev Immunol. (2015) 15:87–103. 10.1038/nri3787 25614319 PMC7162685

[32] Mesev EV LeDesma RA Ploss A. Decoding type I and III interferon signalling during viral infection. Nat Microbiol. (2019) 4:914–24. 10.1038/s41564-019-0421-x 30936491 PMC6554024

[33] Jones SA Jenkins BJ. Recent insights into targeting the IL-6 cytokine family in inflammatory diseases and cancer. Nat Rev Immunol. (2018) 18:773–89. 10.1038/s41577-018-0066-7 30254251

[34] Tanaka T Narazaki M Kishimoto T. IL-6 in inflammation, immunity, and disease. Cold Spring Harb Perspect Biol. (2014) 6:a016295. 10.1101/cshperspect.a016295 25190079 PMC4176007

[35] Turner MD Nedjai B Hurst T Pennington DJ. Cytokines and chemokines: at the crossroads of cell signalling and inflammatory disease. Biochim Biophys Acta. (2014) 1843:2563–82. 10.1016/j.bbamcr.2014.05.014 24892271

[36] Sims JE Smith DE. The IL-1 family: regulators of immunity. Nat Rev Immunol. (2010) 10:89–102. 10.1038/nri2691 20081871

[37] Aggarwal BB. Signalling pathways of the TNF superfamily: a double-edged sword. Nat Rev Immunol. (2003) 3:745–56. 10.1038/nri1184 12949498

[38] Tay MZ Poh CM Rénia L MacAry PA Ng LFP. The trinity of COVID-19: immunity, inflammation and intervention. Nat Rev Immunol. (2020) 20:363–74. 10.1038/s41577-020-0311-8 32346093 PMC7187672

[39] Zlotnik A Yoshie O. The chemokine superfamily revisited. Immunity. (2012) 36:705–16. 10.1016/j.immuni.2012.05.008 22633458 PMC3396424

[40] Matsuura A Ishima T Fujita Y Iwayama Y Hasegawa S Kawahara-Miki R. Dietary glucoraphanin prevents the onset of psychosis in the adult offspring after maternal immune activation. Sci Rep. (2018) 8:2158. 10.1038/s41598-018-20538-3 29391571 PMC5794794

